# Inhaled Carbon Monoxide Provides Cerebral Cytoprotection in Pigs

**DOI:** 10.1371/journal.pone.0041982

**Published:** 2012-08-07

**Authors:** Vicki L. Mahan, David Zurakowski, Leo E. Otterbein, Frank A. Pigula

**Affiliations:** 1 Department of Pediatric Cardiothoracic Surgery, Boston Children’s Hospital, Boston, Massachusetts, United States of America; 2 Department of Surgery, Beth Israel Deaconess Medical Center, Boston, Massachusetts, United States of America; 3 Harvard Medical School, Boston, Massachusetts, United States of America; Charité Universitaetsmedizin Berlin, Germany

## Abstract

Carbon monoxide (CO) at low concentrations imparts protective effects in numerous preclinical small animal models of brain injury. Evidence of protection in large animal models of cerebral injury, however, has not been tested. Neurologic deficits following open heart surgery are likely related in part to ischemia reperfusion injury that occurs during cardiopulmonary bypass surgery. Using a model of deep hypothermic circulatory arrest (DHCA) in piglets, we evaluated the effects of CO to reduce cerebral injury. DHCA and cardiopulmonary bypass (CPB) induced significant alterations in metabolic demands, including a decrease in the oxygen/glucose index (OGI), an increase in lactate/glucose index (LGI) and a rise in cerebral blood pressure that ultimately resulted in increased cell death in the neocortex and hippocampus that was completely abrogated in piglets preconditioned with a low, safe dose of CO. Moreover CO-treated animals maintained normal, pre-CPB OGI and LGI and corresponding cerebral sinus pressures with no change in systemic hemodynamics or metabolic intermediates. Collectively, our data demonstrate that inhaled CO may be beneficial in preventing cerebral injury resulting from DHCA and offer important therapeutic options in newborns undergoing DHCA for open heart surgery.

## Introduction

Neurologic abnormalities occur in neonates undergoing cardiopulmonary bypass (CPB) and deep hypothermic circulatory arrest (DHCA) for correction of congenital heart defects. Neuroprotective strategies have focused on perioperative management with very few innovative options to reduce injury. Preconditioning as a preoperative therapy to protect the brain prior to DHCA has not been studied, but has otherwise been shown to be useful in other clinical indications [1]–[7] such as organ transplantation to improve function post transplant [8]–[13]. The preconditioning agent does not have to be the same as the potentially lethal insult. In organ transplantation, a brief ischemic time elicits a stress response, which leads to altered metabolism, less inflammation, better tissue perfusion and protective gene expression such as the anti-oxidants and heat shock proteins [14]–[21].

Among a host of protective genes is heme oxygenase-1 (HO-1), which is increased dramatically in response to stress and importantly, when elevated, imparts potent salutary effects [22]–[34]. Two isoforms of heme oxygenase exist and are principally responsible for the catalysis of heme into bilirubin. During the breakdown of heme, a molecule of carbon monoxide (CO), biliverdin and iron are released. Biliverdin is rapidly converted to bilirubin by biliverdin reductase while the iron is sequestered into ferritin. The CO generated is exhaled unmodified. Recent efforts have identified CO as the primary mechanism by which HO-1 imparts its beneficial effects in animals.

Inhaled CO is an important therapeutic option and has entered clinical trials (www.clinicaltrials.gov). These include Carbon Monoxide Therapy for Severe Pulmonary Arterial Hypertension, Study of Inhaling Carbon Monoxide to Treat Patients with Intestinal Paralysis after Colon Surgery, and Study of Inhaled Carbon Monoxide to Treat Idiopathic Pulmonary Fibrosis. At low concentrations (15 to 250 ppm), inhaled CO has beneficial effects, mimicking those observed with HO-1 in animal models of ischemia/reperfusion injury, organ transplantation, ileus, necrotizing enterocolitis and airway disease. More recently, Vieira and colleagues showed that CO prevented neuronal apoptosis induced by excitotoxicity and oxidative stress in a primary culture of mouse cerebellar granule cells. [35] In a mouse model, Zeynalov and colleagues evaluated the role of inhaled CO following 90-minutes of transient focal brain ischemia. The authors found that inhalation of 125 ppm or 250 ppm CO begun immediately at the time of onset of reperfusion resulted in reduction in hemispheric infarct volume, improved neurological deficit scores, and limited brain edema. Inhalation of 250 ppm CO begun 1 to 3 hours after ischemia resulted in reduction of infarct volume and improved neurological deficit scores. [36] Wang et al exposed male wild-type and Nrf2-knockout mice to 250 ppm CO or air control for 18 hours immediately after permanent middle cerebral artery occlusion. CO neuroprotection was completely abolished in Nrf2-knockout mice suggesting that the beneficial effect of inhaled CO would at least partially be mediated through the Nrf2 pathway. [37].

However, the role of inhaled CO as a preconditioning neuroprotective agent during DHCA has not been studied. Neuroprotective studies by Mezrow et al suggest that disturbances in cerebral blood flow and cerebral vascular resistance correlate with clinical findings of neurologic injury after hypothermic circulatory arrest and that cerebral metabolism is maintained by increases in oxygen and glucose extraction, a relationship viewed as potentially very important. [38], [39] In order to define effects of inhaled CO on neuroprotection during DHCA, this study investigated differences in cerebral blood flow, cerebral hemodynamics, cerebral metabolism, and cerebral pathology in piglets preconditioned with inhaled CO before undergoing CPB and DHCA.

## Materials and Methods

### Animals

The study was performed at the Animal Research Laboratory Children’s Hospital Boston/Harvard Medical School in Boston and approved by the Institutional Animal Care and Use Committee (IACUC) at Children’s Hospital Boston. All animals received humane care in compliance with the “Principles of Laboratory Animal Care” formulated by the National Society for Medical Research and the “Guide for the Care and Use of Laboratory Animals” published by the National Institutes of Health (NIH Publication No. 88-23, revised 1985).

Twelve Yorkshire female piglets (Parsons Farms, Providence RI), 12 to 19 days old (mean 12.9 days) and weighing 2.2 kg to 4.7 kg (mean 3.4 kg ), were allowed to acclimate 2 days before experimentation with food and water ad libitum. The piglets were randomly assigned to Group I (no preconditioning before CPB/DHCA –6 piglets) or Group II (preconditioned with CO the day before CPB/DHCA –6 piglets).

The Group II animals were preconditioned with inhaled CO (280 ppm CO/balance air – Airgas East, Cambridge MA) 1 day prior to surgery. Piglets were placed in a closed chamber that contained a CO monitoring device (T40 Rattler Single Gas Monitor, Industrial Scientific Corporation, Oakdale PA). The CO gas mixture was pumped into the chamber until a steady state CO concentration of 250 ppm was reached. The piglets were exposed continuously for 3 hours. For safety reasons, the chamber was then flushed with 100% oxygen over 1 minute. The animals were removed from the chamber and returned to their respective pens. Identical exposures were done with controls except the pigs were exposed to normal air.

### Blood Sampling

Baseline blood samples (<1 cc) for all studies was drawn using a blood gas syringe after insertion of the femoral artery catheter and superior sagittal sinus catheter. Repeat samples were drawn during cooling and rewarming on CPB, immediately after weaning from CPB, every 30 minutes after weaning from CPB until death, and when clinically indicated. Blood samples were immediately analyzed for pH, oxygen, carbon dioxide, glucose, lactate, hematocrit, sodium, and potassium using the blood gas analyzer.

### Induction of Circulatory Arrest

On the day of surgery, piglets were premedicated with telazol (6.6 mg/kg), xylazine (2.2 mg/kg), and atropine (0.04 mg/kg) intramuscularly, intubated (non-cuffed 3.0 tube) and placed on isoflourane and the animal’s depth of anesthesia was determined by evaluating reflexes, muscle tone, and response of vital signs to surgical stimulation. They were maintained on positive-pressure ventilation (inspired oxygen greater than 40% and arterial carbon dioxide tension 35 to 45 mm Hg). An intravenous catheter was placed in an ear vein. A nasopharyngeal temperature probe was placed in the esophagus. Femoral artery and femoral venous catheters were placed for monitoring and blood drawing purposes. The animals were placed on a cooling/warming blanket which was initially used to surface cool the animals before DHCA and later to rewarm the animals and to help maintain normal body temperature after DHCA. Cefazolin (25 mg/kg) and methylprednisolone (30 mg/kg) were given intravenously. Animals were surface cooled and the head and groins were packed in ice.

A superior sagittal sinus (sss) cannulation was done prior to cannulation for CPB. A midline scalp incision was made and carried down to the periosteum. The periosteum was removed and a 3 mm cutting burr was used to remove bone over the sinus using 3.5X magnification. A 24 gauge catheter was placed into the sagittal sinus for cerebral venous blood sampling and monitoring of cerebral venous pressure.

A Stockert double roller pump was used to generate nonpulsatile pump flow at an initial rate of 100 ml/kg body weight in all experiments. The oxygenator gas mixture consisted of 5% carbon dioxide and 95% oxygen in all CPB groups. The CPB bypass circuit consisted of the D 901 Lilliput 1 Newborn Hollow Fiber Oxygenator with hardshell venous cardiotomy reservoir (COBE Cardiovascular Inc., Arvada CO), Dideco D736 40 micron Arterial Line Filter (COBE Cardiovascular, Inc., Arvada CO), Custom Smart Tubing Perfusion Pack (COBE Cardiovascular Inc., Arvada CO), Terumo CDI 500 (Terumo Cardiovascular Systems Corp., Tustin CA), and Hemocor HPH 400 (Minntech Corporation, Minneapolis MN). Venous drainage was by gravity. The blood used for priming of the CPB circuit was drawn on the morning of the experiment from an adult donor pig. The CPB circuit was initially primed with Plasmalyte A, sodium bicarbonate 7.4%, 2500 units heparin, and fresh heparinized Yorkshire pig whole blood to achieve a hematocrit greater than or equal to 30%.

Through a median sternotomy, the thymus was reflected superiorly or excised and the pericardium opened. Two hundred U/kg of heparin was given intravenously and the activated clotting time maintained greater than 400 seconds until the piglet was weaned from the circuit. A left atrial catheter for radioisotope injection was positioned through the left atrial appendage into the left atrium. The ascending aorta was cannulated with an 8F Paediatric Arterial Cannula (Polystan A/S, Denmark) and the right atrium was cannulated with a single 12F or 14F venous cannula (Medtronic DLP, Minneapolis MN). A pulmonary artery catheter for monitoring pulmonary artery pressure was placed. CPB was initiated and the flow adjusted to maintain a perfusion pressure of 40 to 50 mmHg. pH-stat strategy (natural alkaline shift in response to the decreasing temperature is corrected by adding carbon dioxide) was used during cooling below 32 degrees Centigrade and for rewarming up to 32 degrees Centigrade nasopharyngeal temperature**.** All animals were cooled using surface cooling and the heater/cooler to a nasopharyngeal temperature of 16 degrees Centigrade and then subjected to 100 minutes of circulatory arrest. Cardiac arrest was achieved after placing a cross-clamp distal to the aortic cannula followed by 20 cc/kg of blood cardioplegia containing 1 part oxygenated pump blood with 4 parts cardioplegia solution (1000 cc plasmalyte, 16.3 cc of 20% mannitol, 4.0 cc of 50% magnesium sulfate, 13.0 cc of 1 mEq/ml sodium bicarbonate, 13.0 cc of 1% lidocaine, and 13.0 cc of 2 mEq/ml potassium chloride) which was administered through a side port of the aortic cannula.

After 100 minutes of circulatory arrest, ice was removed from the head and groins and whole body perfusion was reestablished using the CPB circuit. The animals were rewarmed to a nasopharyngeal temperature of 36 degrees centigrade using the heater/cooler and the cooling/warming blanket. Weaning cardiac support was provided with epinephrine (0.01 to 0.03 µg/kg/minute) and was discontinued in all animals within the first 30 minutes. Weaning from bypass occurred approximately 34 minutes after the start of rewarming. Heparin was reversed with 2 mg/kg intravenous protamine. Animals were maintained on isoflurane anesthesia until they were exsanguinated 6 hours after the termination of CPB.

Piglets were reheparinized with 200 units/kg of heparin 2 minutes before exsanguination. Three liters of chilled normal saline were infused at a pressure of 150 mm Hg through the side port of the clamped aortic cannula followed by 3 liters of 10% formalin. The piglets were decapitated and the head was placed in chilled 10% formalin and refrigerated. Brains were removed *in toto* no sooner than 1 week after death.

### Measurements of Cerebral Blood Flow and Cerebral Vascular Resistance

Cerebral blood flow (CBF) was measured with radionuclide-labeled microspheres as originally described by Rudolph and Heymann. [40] Approximately 0.5 to 1.5×10^6^ microspheres 16±0.5 µm in diameter labeled with Ce141, Cr51, Ru103, Nb95 or Sc46 (PerkinElmer Life and Analytical Sciences, Billerica, MA) were injected and flushed with 5 ml of saline into the left atrial catheter i.) before CPB, ii.) immediately after weaning from CPB, iii.) 1 hour after weaning from CPB, iv.) 3 hours after weaning from CPB, and v.) 6 hours after weaning from CPB. Samples of blood were taken from the femoral artery line at a constant rate (2.00 ml/min) with a Harvard withdrawal pump beginning 15 seconds before injection of microspheres and ending 75 seconds after injection.

### Blood Flow Determination

Fixed brains were bisected in the sagittal plane. Tissue blocks from the left hemisphere were cut to encompass brain regions known for their vulnerability to hypoxia/ischemia. Two specimens were taken from 3 areas of the fixed brain of each animal: frontal neocortex, striatum, and hippocampus. Analyses of these samples to determine blood flow were carried out by computer solution of multiple simultaneous linear equations (Compusphere, Packard instruments). Blood flow was calculated using the following equation: Cerebral blood flow (ml/100 g/min) = (cerebral tissue counts×rate of withdrawal)×100/(counts in reference sample×weight of brain sample). Cerebral vascular resistance (CVR) was calculated using the equation: CVR (mm Hg/ml/100 g/min)  =  [MAP – MSSSP (mm Hg)]/CBF (ml/100 gm/min) where MAP is mean arterial pressure and MSSSP is mean superior sagittal sinus pressure.

### Assessment of Cell Death

Damaged neurons (DN) included nuclear pyknosis with wrinkled nuclear outlines and minimal cytoplasmic change, nuclear pyknosis with eosinophilic cytoplasm, ghost neurons, or apoptotic bodies. Counts were scored as follows: 1, 0 to 5 DN seen per high-power field; 2, 6 to 15 DN seen per high-power field; 3, 16 to 25 DN seen per high-power field; and 4, greater than 25 DN seen per high-power field. Twenty randomly selected 400× fields of H&E stained frontal neocortex, striatum, and hippocampus were examined for each animal by bright-field microscopy. Counts for each area were averaged for a final score.

### Serum Measurements

Blood samples from the femoral artery and superior sagittal sinus were obtained simultaneously for calculation of cerebral oxygen extraction (arteriovenous difference in content of oxygen), cerebral glucose extraction (arteriovenous difference in glucose), cerebral lactate extraction (arteriovenous difference in lactate), oxygen/glucose index (OGI), and lactate/glucose index (LGI).

### TUNEL Staining and Quantitation

Tissue sections from the right frontal neocortex/striatum and hippocampus were cut from paraffin blocks at a thickness of 5 µm and mounted. Sections were stained by terminal nick-end labeling of cleaved DNA with fluorescein-conjugated nucleotides (ApopTag Fluorescein In Situ Apoptosis Detection Kit; Chemicon International, Billerica, Massachusetts). The sections were also stained for nuclei (DAPI; Molecular Probes, Eugene, Oregon). TUNEL-positive and –negative nuclei were counted using Metamorph software (Version 6.2; Molecular Devices, Downingtown, Pennsylvania) on 5 to 10 random frontal neocortex/striatum and hippocampus fields per section, read at low magnification. Results are expressed as apoptotic nuclei (AN) per 1,000 total nuclei.

### Caspase-3 Immunohistochemistry

Formalin-fixed, paraffin-embedded brain tissue sections of hippocampus were deparaffinized with xylene, rehydrated gradually with graded alcohol solutions (100%, 95%, and 80%), and then washed with deionized water. For antigen unmasking, sections were treated in trypsin solution for 10 minutes at 37°C. Sections were then washed with deionized water and incubated with 3% H_2_O_2_ for 5 minutes. Sections were then incubated with a 1∶300 dilution of the rabbit polyclonal anti-active caspase-3 (Promega, Madison, WI) overnight at 4°C. After three PBS washes, sections were incubated with the secondary antibody, a biotinylated goat anti-rabbit IgG, at 37°C for 30 minutes, and with peroxidase-conjugated strepavidin-biotin complex (Santa Cruz Biotechnology, Santa Cruz, CA) at 37°C for 30 minutes. Diaminobenzidine (DAB) substrate (Zymed, South San Francisco, CA) was applied as the chromogen, giving a brown reaction product, and the sections were counterstained with Mayer’s hematoxylin.

### Statistical Analysis

The Kolmogorov-Smirnov goodness-of-fit test was used to assess for normality of the data and no gross departures were detected. Therefore, baseline values were compared by one-way analysis of variance (ANOVA) with means and standard deviations reported. Mean differences between the treatment groups at various time points were assessed using two-way repeated-measures ANOVA (group and time as factors) with a mixed-model approach and a compound symmetry covariance structure to account for the within-animal correlation. [41] The Greenhouse-Geisser F-test for small samples was chosen for assessing significance group and time effects. [42] TUNEL results were compared between the two groups using a Poisson regression model for analyzing count data. Analysis of the data was performed with use of the PROC MIXED and GEE procedures in the SPSS software package (version 16.0, SPSS Inc./IBM, Chicago, IL). Two-tailed values of *P*<.05 were regarded as statistically significant.

## Results

### Hemodynamic and Hematologic Parameters Remained Stable Throughout CPB

Preoperative data (age and weight) were similar. Cooling and rewarming times during CPB were similar. There were no statistically significant differences at baseline between the groups regarding temperature, mean arterial pressure, arterial blood gases, sss blood gases, sss saturation values, oxygen extraction ratio, hematocrit values, arterial glucose values, and arterial lactate values, which were compared using 2-way repeated-measures ANOVA. **(** **Table 1** **)** No significant differences between the 2 groups were found for any of these variables throughout the time period from baseline to 6 hours after weaning from CPB.

**Table 1:** Hemodynamic and Hematologic Parameters.

	PRE CPB	AFTER CPB	1 HR AFTER CPB	3 HRS AFTER CPB	6 HRS AFTER CPB
TEMP					
CONTROL	32.4+/−1.6	34.6+/−1.4	32.3+/−2.0	34.2+/−1.1	35.9+/−1.3
CO-TREATED	33.0+/−2.1	34.0+/−2.6	33.1+/−1.8	35.9+/−1.8	36.2+/−1.2
MAP (mm Hg)					
CONTROL	55+/−10	74+/−14	66+/−6	65+/−8	60+/−10
CO-TREATED	54+/−8	71+/−13	62+/−6	59+/−4	58+/−4
pHa					
CONTROL	7.49+/−0.10	7.36+/−0.11	7.43+/−0.04	7.45+/−0.06	7.43+/−0.06
CO-TREATED	7.50+/−0.06	7.40+/−0.06	7.40+/−0.04	7.44+/−0.06	7.42+/−0.08
PaO2 (mm Hg)					
CONTROL	619+/−67	314+/−184	451+/−199	444+/−142	441+/−171
CO-TREATED	600+/−106	372+/−279	463+/−162	419+/−142	480+/−183
PaCO2 (mm Hg)					
CONTROL	37.7+/−9.7	46.3+/−13.9	40.2+/−4.1	41.9+/−4.8	43.5+/−3.3
CO-TREATED	34.6+/−8.5	38.3+/−10.7	40.1+/−4.8	39.1+/−0.9	42.1+/−5.0
pHsss					
CONTROL	7.42+/−0.08	7.30+/−0.10	7.34+/−0.01	7.38+/−0.04	7.35+/−0.06
CO-TREATED	7.43+/−0.05	7.36+/−0.03	7.31+/−0.05	7.35+/−0.06	7.38+/−0.03
PsssO2 (mm Hg)					
CONTROL	44.1+/−11.8	50.6+/−10.5	37.9+/−6.6	46.3+/−8.8	47.4+/−15.8
CO-TREATED	45.3+/−13.4	43.4+/−18.6	44.3+/−9.9	39.3+/−9.9	43.4+/−12.2
PsssCO2 (mm Hg)					
CONTROL	48.2+/−12.4	56.6+/−13.6	60.0+/−9.8	55.7+/−6.1	58.4+/−6.5
CO-TREATED	46.9+/−5.4	47.4+/−7.2	55.5+/−6.7	57.2+/−6.7	53.3+/−5.2
sss Saturation (%)					
CONTROL	60+/−10	64+/−14	50+/−12	59+/−12	60+/−22
CO-TREATED	69+/−19	61+/−27	56+/−13	51+/−14	60+/−15
OER					
CONTROL	70+/−6	53+/−11	66+/−18	62+/−14	63+/−16
CO-TREATED	63+/−10	58+/−18	65+/−10	66+/−14	67+/−11
HEMATOCRIT					
CONTROL	35.2+/−4.5	36.7+/−5.8	34.7+/−5.6	35.3+/−8.1	34.2+/−7.6
CO-TREATED	37.3+/−8.3	38.2+/−4.3	35.7+/−2.4	37.8+/−5.0	33.0+/−4.2
Arterial Glucose					
CONTROL	161+/−58	138+/−36	142+/−51	181+/−53	201+/−58
CO-TREATED	143+/−122	136+/−81	173+/−78	174+/−83	184+/−57
Arterial Lactate					
CONTROL	4.0+/−2.8	9.9+/−4.3	7.4+/−1.7	5.2+/−1.7	4.6+/−3.9
CO-TREATED	3.5+/−1.3	8.2+/−1.3	7.5+/−0.9	5.7+/−1.8	5.4+/−1.7

### Effects of Inhaled CO on Systemic and Cerebral Hemodynamics

**Figure 1:**
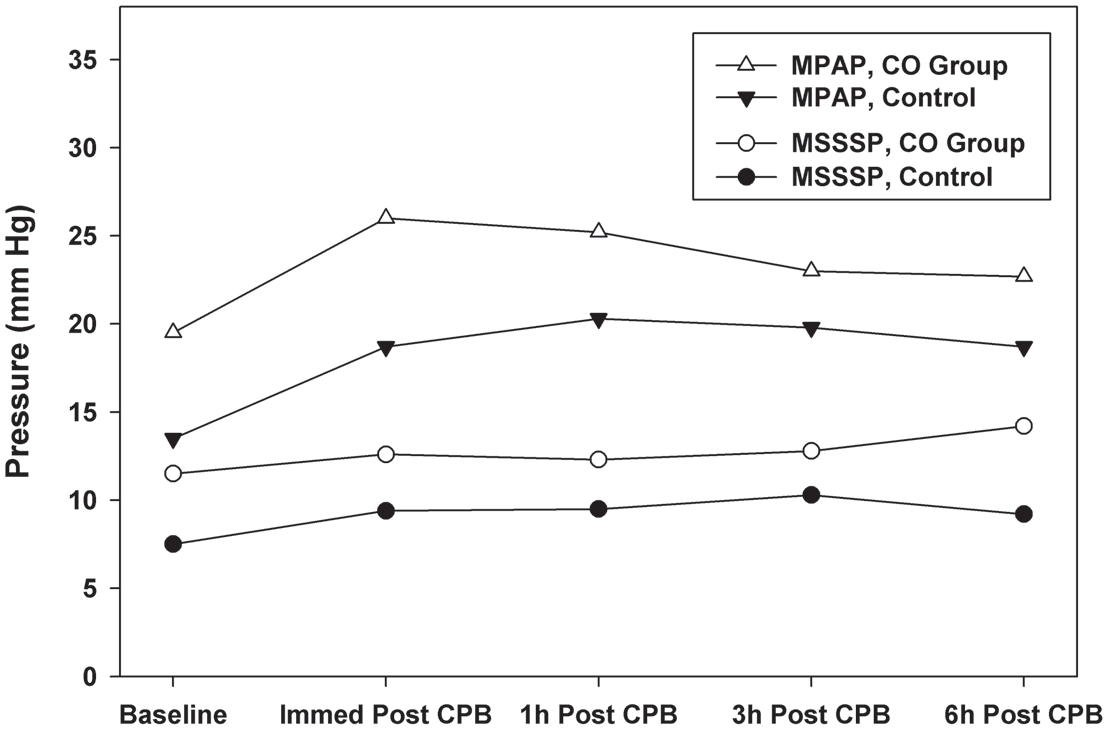
Mean pulmonary artery pressure (MPAP) and mean superior sagittal sinus pressure (MSSSP) were higher in the CO preconditioned piglets at all time points. Data represent the means ± SD of 6 animals/group, MPAP p<0.05 immediately after CPB and MSSSP p<0.05 6 hours after CPB.

**Figure 2:**
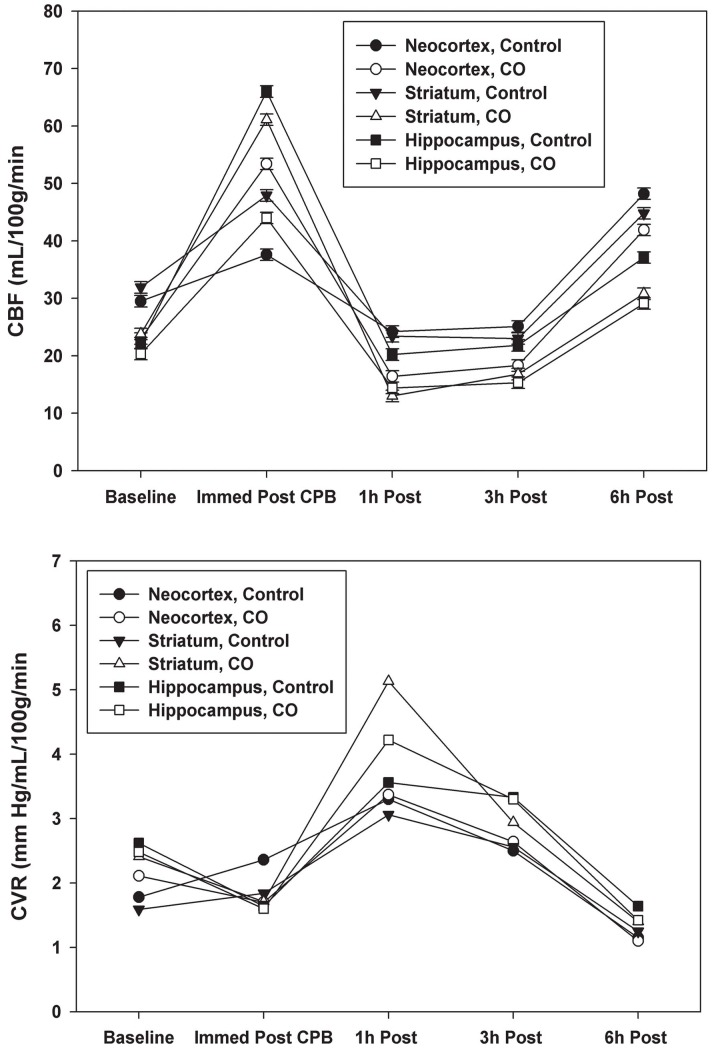
(CBF). Data represent the means ± SD of 6 animals/group. Cerebral blood flow (CBF) was similar in air and CO-treated piglets at all time points. After weaning from CPB, CBF increased from baseline and then decreased to below baseline at 1 and 3 hours after weaning. At 6 hours after weaning, CBF was above baseline. There were no statistical differences between the Air and CO groups. **2 (CVR).** Data represent the means ± SD of 6 animals/group. Cerebral vascular resistance (CVR) reflected changes seen with CBF and were similar in air and CO-treated piglets at all time points. CVR was higher than baseline in both groups 1 hour and 3 hours after weaning from CPB and below baseline 6 hours after weaning. There were no statistical differences between the Air and CO groups.

Mean pulmonary artery pressure (MPAP) and mean superior sagittal sinus pressure (MSSSP) were higher in CO preconditioned animals at all time points (***Figure 1***). Statistical significance was observed for MPAP immediately after CPB and for MSSSP 6 hours after CPB. Although cerebral perfusion pressure (MAP – MSSSP) was slightly higher in the control group during the study, this difference did not reach statistical significance. Cerebral blood flow (CBF) was similar in both air and CO-treated piglets at all time points (***Figure 2***). Immediately after weaning from CPB, CBF increased markedly from baseline and then decreased to below baseline at 1 hour and 3 hours after weaning from CPB. Six hours after weaning from CPB, CBF was higher than baseline in all groups and was similar in air and CO-treated piglets.

Cerebral vascular resistance (CVR) was elevated over baseline in both groups 1 hour and 3 hours after weaning from CPB and then dropped below baseline 6 hours after weaning. This was expected based on CBF (***Figure 2***). CVR immediately after weaning did not change significantly from baseline with no statistical difference between air or CO-treated animals.

Given the reports that CO can impart vasodilatory effects, we expected that cerebral perfusion pressures and CBF would be higher and that CVR would be lower than what was observed in the air treated animals. These results suggest that preconditioning with CO was ineffective at modulating cerebral vasodilation and improved cerebral blood flow immediately before or after CPB.

### CO Influences Metabolism in the Brain Following CPB

**Figure 3:**
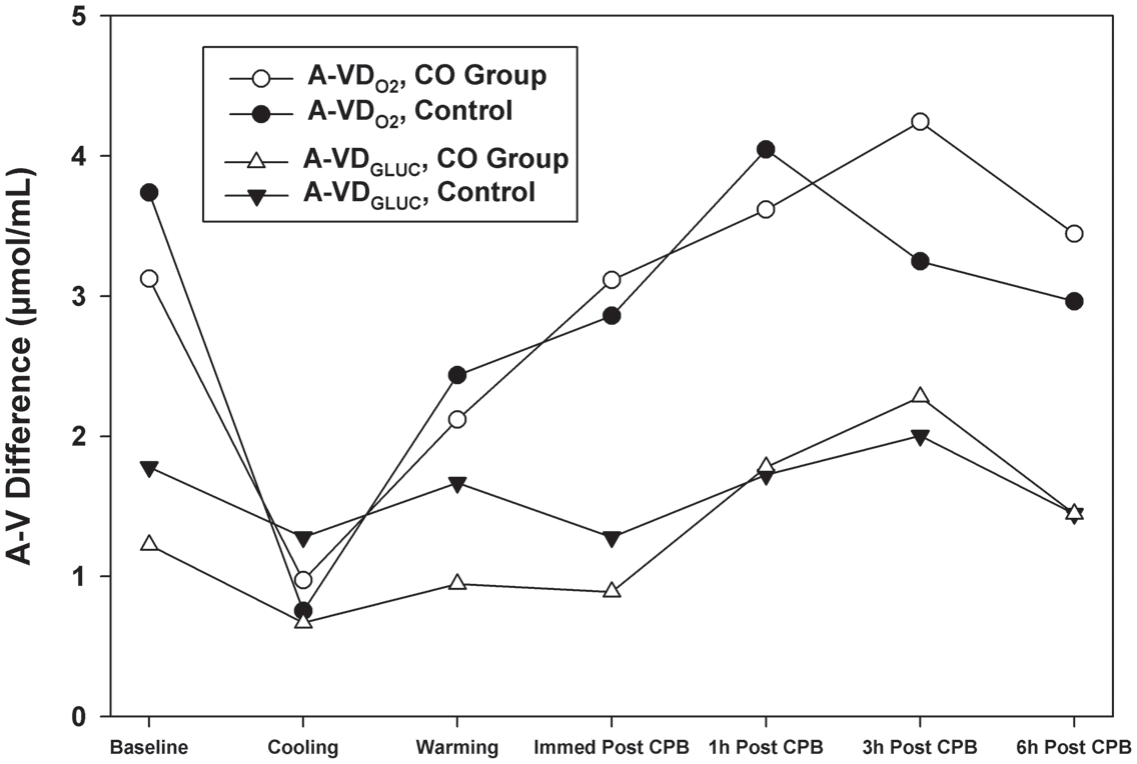
Arteriovenous difference in oxygen tension (A-VD_O2_) was greater than arteriovenous difference in glucose (A-VD_GLUCOSE_) except during cooling when A-VD_GLUCOSE_ of the control animals was greater than A-VD_O2_. A-VD_O2_ in the CO-treated group was higher at 3 and 6 hours after CPB. A-VD_GLUCOSE_ was higher in the air group at baseline, during cooling and rewarming on CPB and immediately after CPB. While there were trends in A-VD_O2_ and A-VD_GLUCOSE_ values between air and CO-treated animals, no significant differences were observed. Data represent the means ± SD of 6 animals/group.

Differences between arteriovenous oxygen tension (A-VD_O2_) were greater than differences between arteriovenous glucose (A-VD_GLUCOSE_) at baseline. No significant differences between A-VD_O2_ and A-VD_GLUCOSE_ values between air and CO-treated animals were observed. (***Figure 3***).

The oxygen/glucose index (OGI) describes the relationship between A-VD_O2_ and A-VD_GLUCOSE_ and is independent of cerebral blood flow**.** Stoichiometrically, 6 moles of oxygen are required to metabolize 1 mole of glucose. If all glucose is oxidized and no other substrate is consumed, A-VD_O2_/A-VD_GLUCOSE_ is equal to 6. The ratio is less than 6 if all the glucose extracted is not oxidized and greater than 6 if other substrates (i.e. lactate, pyruvate, acetoacetate, β-hydroxybutyrate, glutamate) are used as a source of carbon. The equation for OGI defines this biochemical relationship:
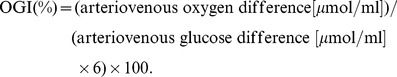



If all of the glucose extracted is oxidized and no other substrate is consumed, OGI(%) is 100%. Values less than 100% indicate that not all of the glucose extracted is oxidized (anaerobic metabolism) and values greater than 100% indicate that other substrates are used as a source of carbon.

**Figure 4:**
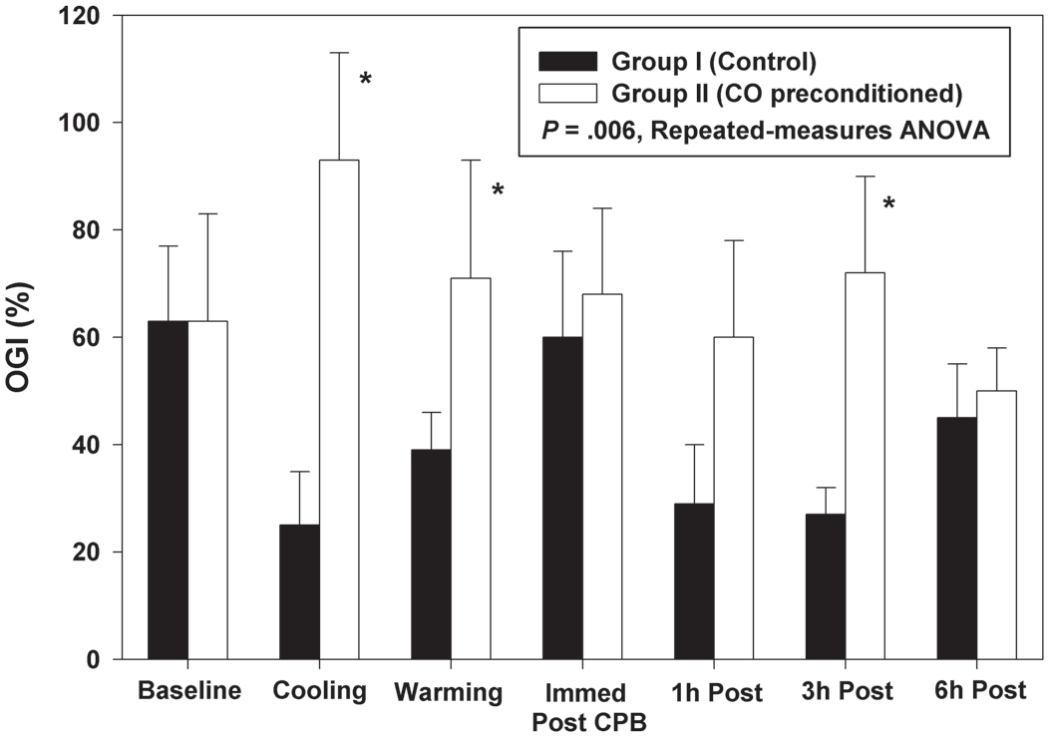
OGI(%) was similar at baseline in both groups, but was above baseline in the CO-treated group thereafter. In the air group, OGI(%) was below baseline at all time points. Data represent the means ± SD of 6 animals/group, p = 0.006 repeated measure ANOVA. * indicates statistical significance.

Prebypass mean OGI (%) was comparable in both groups and determined to be 63% (±42) in the control group and 63% (±47) in the CO group. The OGI was higher in the CO-treated animals at all other time points (p = 0.006; ***Figure 4*** , * indicates statistical significance).

In the control group the OGI decreased with CPB and cooling. This is attributed to a reduction in A-VD_O2_ to a greater extent than the reduction in A-VD_GLUCOSE_ (***Figure 3***). During rewarming, the OGI returned to baseline immediately after weaning from CPB. OGI then decreased to below baseline values for the duration of the study. (***Figure 4***).

In the CO-treated group, the OGI increased over baseline in the presence of CPB and cooling. (***Figure 4***) A-VD_O2_, superior sagittal sinus saturation and oxygen extraction ratios were in close agreement between the groups, but A-VD_GLUCOSE_ was lower in the CO group before, during and immediately after CPB (***Figure 3***) suggesting that glucose was being metabolized at a lower rate in the CO-treated animals. Both A-VD_O2_ and A-VD_GLUCOSE_ returned to baseline by the end of the study. The calculated OGI, likewise, returned to baseline.

**Figure 5:**
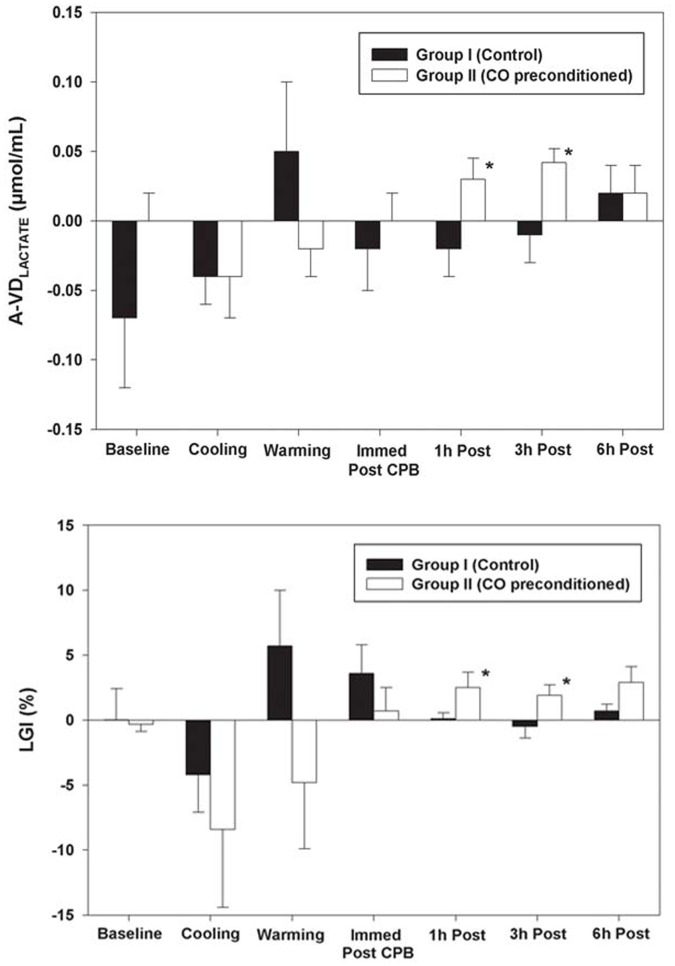
(A-VD_LACTATE_). In the air group, A-VD_LACTATE_ was more negative at baseline than the CO-treated group, became positive during warming on CPB, and was negative immediately after weaning from CPB, 1 hour after CPB, and 3 hours after CPB. In contrast, A-VD_LACTATE_ was negative during CPB and become positive after weaning from CPB. At 6 hours after CPB, A-VD_LACTATE_ was positive in both groups. Data represent the means ± SD of 6 animals/group, p<0.05 at 1 hour after CPB and 3 hours after CPB. * indicates statistical significance. **5 (LGI%).** LGI% was similar at baseline and negative in both groups during cooling on CPB. In the air group, LGI% became positive during warming on CPB. LGI% was negative in the CO_treated animals during warming on CPB but was positive thereafter. Data represent the means ± SD of 6 animals/group, p<0.05 at 1 hour after CPB and 3 hours after CPB. * indicates statistical significance.

A negative A-VD_LACTATE_ is consistent with lactate being produced in the brain. Assessment of A-VD_LACTATE_ levels trended toward being more negative in the control group basally, but did not achieve statistical significance (***Figure 5***). During cooling on CPB, A-VD_LACTATE_ levels were similar. While A-VD_LACTATE_ was negative in air and CO-treated animals during cooling, A-VD_LACTATE_ became positive in the control group and remained negative in CO-treated animals during rewarming on CPB. After weaning from CPB, however, A-VD_LACTATE_ became negative in controls and remained low in contrast to CO-treated animals which recovered and were positive after CPB at 1 and 3 hr (1 hr post CPB: air at −0.02 µml/ml versus CO at +0.03 µmol/ml, p<0.05; 3 hr post CPB: air at −0.01 µmol/ml versus CO at +0.04 µmol/ml, p<0.05) at which point the A-VD_LACTATE_ in both groups became positive ***(*** ***Figure 5*** **,** * indicates statistical significance).

The lactate/glucose index (LGI) is a quantitative expression describing the amount of glucose consumption that appears as lactate production. If all extracted glucose is metabolized to lactate, A-VD_LACTATE_/A-VD_GLUCOSE_ is equal to the absolute value 2. The equation for LGI defines this biochemical relationship:
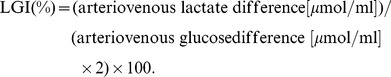



If all of the extracted glucose is metabolized to lactate the LGI (%) is 100%. A negative LGI results if glucose is metabolized to lactate (sss lactate is higher than arterial lactate.) A positive LGI suggests that lactate is further metabolized by the cell and tissue.

The LGI basally before surgery were 0.03 and −0.25 for the control and CO-treated animals respectively with both air and CO being negative during cooling with no statistical difference between groups observed. The LGI became positive in control animals (1.25) during rewarming on CPB suggesting that lactate was metabolized by the brain during this time (***Figure 5*** , * indicates statistical significance). The LGI in the CO-treated animals was negative (−4.80) during rewarming, but became positive in CO-treated animals immediately after weaning from CPB (0.69) Differences between air treated and CO-treated groups reached statistical significance 1 hour post CPB (air treated group 0.15 and CO-treated group 2.50, p<0.05) and 3 hours post CPB (air treated group −0.48 and CO-treated group 1.92, p<0.05).

Taken together these data suggest that CO induces a metabolic change in the brain of piglets undergoing CPB/DHCA. OGI was higher in CO-treated animals except at baseline and was statistically significant during cooling and warming on CPB and at 3 hours after weaning from CPB. One hour after weaning from CPB, LGI remained higher in CO-treated animals and was statistically significant at 1 hour and 3 hours after weaning from bypass.

### CO Abrogated Apoptosis of Neurons

**Figure 6:**
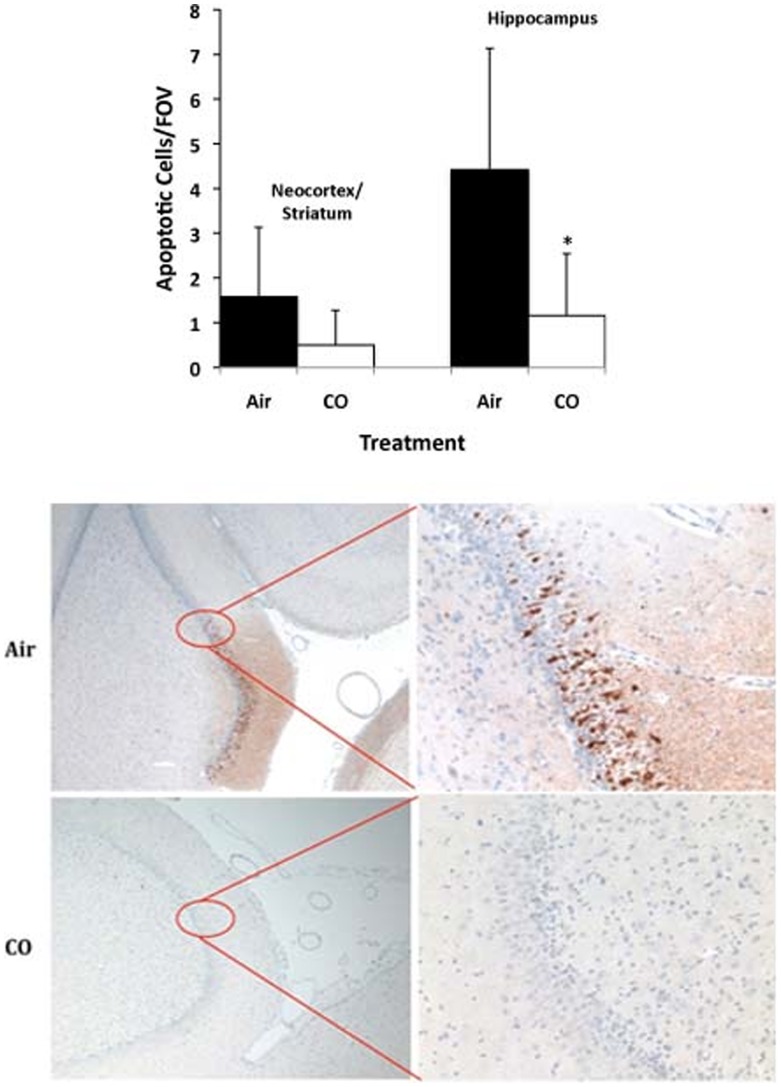
(TUNEL). Quantitation of TUNEL staining in indicated brain sections from neonatal piglet after CPB ± CO showed more apoptosis in the control group versus CO in the hippocampus and trending sections from the neocortex and striatum. Data represent mean ± SD of 6 animals/group, *p<0.03. Untreated pigs showed <0.1 positively stained cell/field of view (FOV). **(Caspase-3).** Representative tissue sections stained for activated Caspase-3 in the hippocampus from neonatal piglets after CPB ± CO. Note the intense positive brown staining indicating activated caspase-3 localized to the hippocampus in the control, air-treated pigs versus nearly no positive staining in the CO treated pigs. Images are representative of n = 6–8/group.

Cellular damage in the frontal neocortex, striatum, and hippocampus was determined by TUNEL staining. Cell death likely comprised a combination of necrosis and apoptosis in both groups (***Figure 6***). TUNEL staining showed a significant increase in positive apoptotic counts in the neocortex/striatum versus naïve which was abrogated in CO-treated piglets versus controls (0.5/1000 cells versus 21.6/1000 cells), but did not achieve significance in this cohort. In the hippocampus, however, there was a greater number of TUNEL positive cells in the air-treated animals which was significantly reduced counts in CO-preconditioned piglets (4.4/1000 cells versus 1.1/1000 cells respectively, p<0.03). Caspase-3 staining of the hippocampus showed a greater number of caspase-3 positive staining, indicative of cell death in air-treated animals undergoing –CPB and DHCA. In contrast, animals treated with CO and then subjected to CPB and DHCA showed a marked reduction in caspase-3 positive staining in the hippocampus (***Figure 6***) thereby indicating that CO offers salutary effects in limiting cell death in the brain in piglets undergoing CPB and DHCA.

## Discussion

The data presented here continue to support the concept that low concentrations of inhaled CO are beneficial in models of ischemic injury. A clinically relevant CO preconditioning regimen imparted potent cerebroprotection evidenced by less cell death in the cortex and hippocampus in response to CPB and DHCA. While the exact molecular target remains unknown, there is clearly an effect of CO that results in altered bioenergetics and metabolism in the brain.

Cerebroprotective effects have been well-described with HO-1 induction. [43]–[45] The protective effects of HO-1 induction, and therein the endogenous generation of CO, can be mimicked in nearly all instances using inhaled CO or administration of carbon monoxide releasing molecules (CO-RMs). [46] Activation of HO-1 or administration of CO has been shown to regulate a number of specific cell signaling pathways including MAP kinases, BKCa channels, guanylyl cyclase, PPARγ, and HIF1α. [47] Modulation of these pathways is highly dependent on when CO is administered, either pre or post stressor. Paradoxically, CO increases ROS generation when administered as a pretreatment primarily through binding to mitochondrial oxidase complexes. The transient ROS generation leads to preconditioning of the cell towards an anti-inflammatory and anti-apoptotic phenotype via upregulating cytoprotective molecules including HO-1.

Our study suggests that a change in metabolic substrate modulation may be key in neuroprotection. Functions of the central nervous system are mainly excitation and conduction, electrical energy being derived from chemical processes. Stoichiometric modeling of brain metabolism in response to tissue hypoxia involves central metabolism (glycolysis, pentose phosphate pathway, TCA cyle), lipid metabolism, reactive oxygen species detoxification, amino acid metabolism, the glutamate-glutamine cycle and neurotransmitter metabolism. [48] There has been continued debate as to the primary fuel of brain cells during basal and stressed conditions. [49] Lactate may be the preferred energy substrate of activated neurons. Our findings are consistent with a change to lactate as the metabolic substrate for neurons in a piglet model of DHCA/CPB. A-VD_LACTATE_ and LGI became positive and statistically significant at 1 and 3 hours after weaning from CPB. Changes in bioenergetics in response to CO may likewise reflect the observed cytoprotective effects. Historically, this would corroborate the fact that CO targets the mitochondrial hemoproteins to modulate ATP generation and O_2_ consumption; however this was performed in isolated mitochondria and not whole cell preparations or tissues. CO also activates guanylate cyclase to increase cGMP levels in neurons and subsequent opening of mitochondrial K channels, critical for CO inhibition of apoptosis. [35] Queiroga and colleagues studied the ability of CO to prevent apoptosis in primary astrocytes and observed that the antiapoptotic effects of CO is due to the inhibition of mitochondrial membrane permeabilization, a major factor in the intrinsic apoptotic pathway. [50] Precisely how metabolism of lactate relates to the changes observed in mitochondria and subsequent cerebral cytoprotection will help to further define the role of this metabolic substrate as a neuroprotective agent. We speculate that lowering cellular dependency on oxidative phosphorylation, even transiently, would lead to reduced ROS and be reflected as abrogated cell death.

CO also acts to influence vasomotor tone, albeit rather small potency when compared to nitric oxide, but has been shown regulates cerebral vasodilation. [51] Exogenous administration of CO dilates pial arteries of baby and juvenile pigs acting as an autocrine/paracrine messenger in the regulation of cerebrovascular hemodynamics and can prevent epileptic seizures in newborn piglets with no loss of cerebrovascular reactivity 2 days after administration of CO. Zimmermann et al concluded that pretreatment of the piglets elicits vasodilator properties on the cerebral circulation protecting the brain from cerebrovascular injury caused by seizures. [52] Immediately following DHCA, an elevated intracranial pressure (ICP) may result in reduction of cerebral blood flow and it is speculated that neurophysiological recovery and behavior are impaired in animals with a higher ICP and that suppression of cerebral metabolism is associated with a lower ICP. [53] Daley and colleagues showed that this preconditioning effect results in increased ICP caused by cerebral vasodilation in the piglet model. [54] In hypoxia/ischemia injury seen with DHCA, elevation of ICP is associated with brain pathology [55], [56]. In our study, superior sagittal sinus saturation and superior sagittal sinus oxygen content were similar in Groups I and II (air vs CO respectively). Cerebral perfusion pressures and CBF and CVR in the neocortex, striatum, and hippocampus were also similar between treatment groups. CO-treated animals exhibited higher MSSSP with less apoptosis which did not correlate with decreased oxygen delivery, changes in CBF or neuropathology. Mean pulmonary artery pressures (MPAP) were higher in the CO-treated group and statistically significant immediately after CPB. The higher MPAP seen in the CO-treated animals may explain the elevated MSSSP observed in the CO treated animals; however, cerebral perfusion pressures and CBF and CVR in the neocortex, striatum, and hippocampus were similar between treatment groups. These results suggest that preconditioning with CO modulates cerebral injury following a prolonged period of DHCA despite an increase in MSSSP and might improve cerebral outcome after a prolonged period of DHCA despite an increased MSSSP. The observed cerebroprotective effects may result from changes in cerebral metabolism or modulation of inflammation and apoptosis which is the focus of future experiments [57]. Additionally, in a pig model of kidney transplant and ischemic-reperfusion injury, CO was also protective when administered intraoperatively [58]. CO treatment of recipients only led to increased repair of the damaged kidney through induction of proliferative genes. This is unlikely the mode of action of CO here since CO was administered as a pretreatment, but remains to be explored. More likely is that the preconditioning results in a state of tolerance to the subsequent stress.

Cerebral metabolism with DHCA is highly complex and incompletely understood. Metabolic compartmentalization in the intact brain reflects metabolic alterations between different neuronal components and even among different neuronal populations. Under normal physiological conditions, glucose is the primary source of fuel for the brain. The distribution of energy substrates from the systemic circulation into neurons is principally determined by astrocytes and the dependence of cerebral function on blood glucose as a fuel does not exclude lactate or other substrates as an energy source. The concept of “coupling” of oxidative metabolism and functional activity in the brain has, however, been challenged. A greater increase in CBF and CMR_GLUCOSE_ with little change in CMR_O2_ during stimulation suggest increased lactate production as active neurons release large amounts of lactate [59]–[61]. In a seizure model in rats, Schridde et al showed that increases in hemodynamic, metabolic, and neuronal activity are dependent on the interaction between hemodynamics and metabolism. [62].

During hypoxia and ischemia, astrocytes provide both structural and metabolic support and exhibit a high glycolytic rate generating primarily lactate. Vega and colleagues concluded that there is a shift in the metabolic response to energy conservation and a change in cerebral metabolism [63]. Bouzier and colleagues showed that ^13^C-labeled lactate was a major substrate for oxidative metabolism in C6 glioma cells and hypoxic conditions were found to lead to the accumulation of lactate as a rich energy source. [64] In a study in newborn piglets with intrauterine growth restriction, Moxon-Lester et al concluded that increased cerebral lactate is neuroprotective. [65] In our study, A-VD_O2_ and A-VD_GLUCOSE_ showed no differences between air- and CO-treated animals, but statistically significant differences in OGI (statistically significant during cooling and warming and 3 hours after weaning from CPB), A-VD_LACTATE_ (statististically significant 1 hour and 3 hours after weaning from CPB), and LGI (statistically significant 1 hour and 3 hours after weaning from CPB) between groups. The change in glucose and lactate metabolism in the brains of piglets preconditioned with CO undergoing CPB/DHCA showed less lactate production and/or higher lactate consumption. Figure 4 suggests that CO increases oxidative metabolism which would result in less lactate production. However, higher lactate consumption by neurons induced by CO as an alternative source of carbon cannot be overlooked.

Schurr et al and Occhipinti et al studied the role of lactate during glycolysis and hypothesized that lactate is the primary energy substrate for neurons under aerobic conditions and that interference with neuronal lactate utilization could result in damage [66], [67]. Gordon et al showed that the ability of astrocytes to induce vasodilation relies on the metabolic state of rat brain tissue and that lactate attenuates transporter-mediated uptake of prostaglandin E2 resulting in vasodilation, a preconditioning effect [68]. Won and colleagues have investigated whether intraperitoneal injection of lactate after hypoglycemia reduced neuronal death. Lactate administration with glucose supplementation reduced neuronal death by 80% in the hippocampus and reduced superoxide production and microglia activation. The authors hypothesize that increasing brain lactate following hypoglycemia offsets the decrease in NAD(+). This was found to be due to overactivation of PARP-1 acting as an alternative energy substrate that bypassed glycolysis and be fed directly to the citric acid cycle to maintain cellular ATP levels. [69] In our study, A-VD_LACTATE_ and LGI became positive in both groups, but was more striking in the CO preconditioned piglets. This suggests a change in cerebral metabolism of lactate related to CO preconditioning prior to DHCA that may be important in neuroprotection.

In summary, preconditioning of the neonatal piglet with inhaled CO prior to CPB/DHCA did not result in differences in CBF and CVR between groups. MSSSP was increased in the preconditioned animals, but A-VD_O2_ was not changed. There were significant differences in glucose and lactate metabolism between the groups and corresponding histopathology clearly showed that apoptosis was significantly less in the CO-treated animals. These results suggest that preconditioning with CO results in a change in cerebral metabolism of glucose and lactate that corresponds with neuroprotection. Whether the change in tissue bioenergetics is a direct effect of CO or indirectly through improved tissue protection remains to be determined. With CO currently in clinical trials, application of these findings towards patients undergoing CPB may be imminent.
